# Supplementary material for: Antibiotic discovery with artificial intelligence for the treatment of Acinetobacter baumannii infections
Source: mSystems. 2024 May 3;9(6):e00325-24. doi: 10.1128/msystems.00325-24 (PMC11326114; doi:10.1128/msystems.00325-24)
Supplement: Figure S1 — 3D structure and protein sequence of OmpW. [file msystems.00325-24-s0001.pdf]

SUPPLEMENTARY MATERIAL

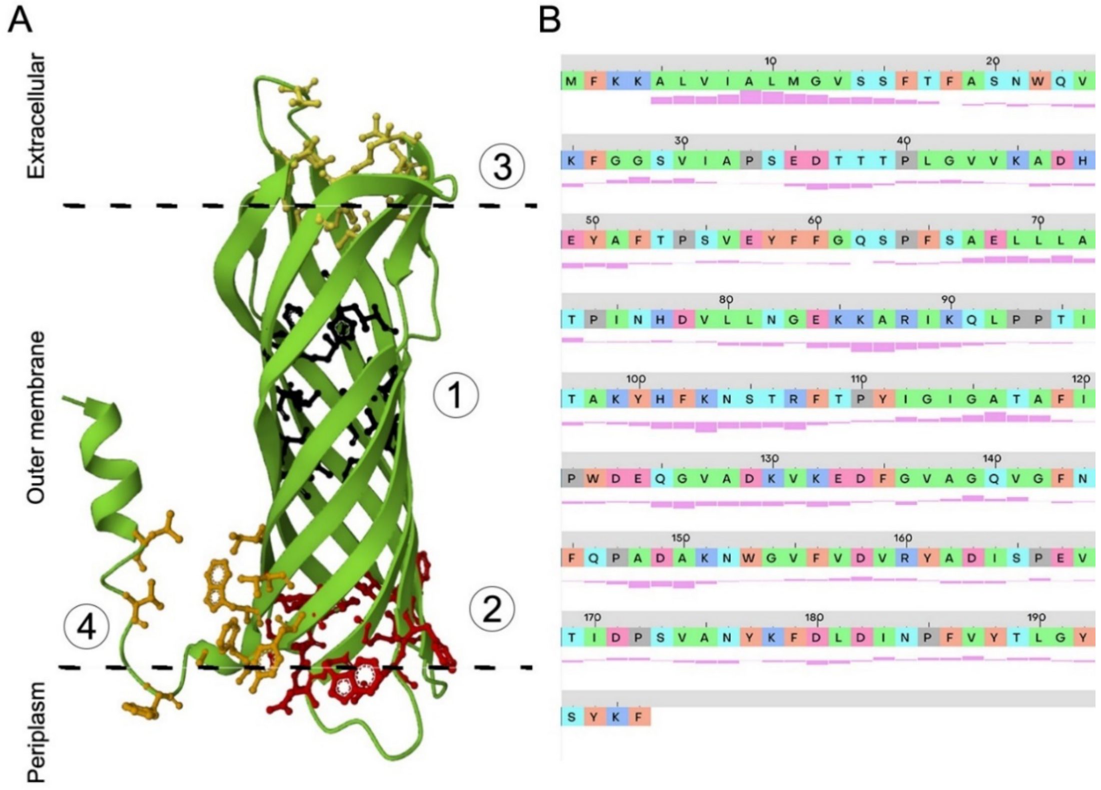

Figure S1. 3D structure of OmpW with each predicted binding pocket (**A**). Protein sequence of OmpW with the hydrophobicity level of each amino acid (**B**).
