# Supplementary material for: Antibiotic discovery with artificial intelligence for the treatment of Acinetobacter baumannii infections
Source: mSystems. 2024 May 3;9(6):e00325-24. doi: 10.1128/msystems.00325-24 (PMC11326114; doi:10.1128/msystems.00325-24)
Supplement: Table S1 — Predicted binding pockets for OmpW. [file msystems.00325-24-s0002.pdf]

# SUPPLEMENTARY MATERIAL

**Table S1**. Predicted binding pockets for OmpW.

| Name | Score | Probability | Center_x | Center_y | Center_z | Residue_ids |
|---|---|---|---|---|---|---|
| pocket1 | 12.87 | 0.627 | -2.82 | -0.55 | 1.61 | A_117 A_119 A_138 A_160 A_162 A_164 A_183 A_185 A_187 A_31 A_53 A_70 A_77 A_91 A_93 A_95 |
| pocket2 | 8.69 | 0.443 | 10.39 | -0.52 | -17.85 | A_101 A_102 A_103 A_104 A_106 A_109 A_144 A_145 A_146 A_152 A_153 A_154 A_193 A_195 A_23 A_59 A_61 A_63 A_64 |
| pocket3 | 6.36 | 0.287 | -15.81 | -3.1 | 17.51 | A_127 A_128 A_168 A_170 A_174 A_179 A_39 A_41 A_44 A_79 A_81 A_86 A_87 |
| pocket4 | 2.41 | 0.052 | 6.91 | 15.69 | -15.66 | A_10 A_13 A_19 A_196 A_22 A_24 |
